# Errors in Abstract, Statistical Analysis, Results, Tables 1, 2, and 3, and Supplement 1

**DOI:** 10.1001/jamanetworkopen.2026.34722

**Published:** 2026-08-27

In the Original Investigation titled “Procalcitonin to Guide 7 vs 14 Days of Antibiotics in Bloodstream Infections: A Secondary Analysis of the BALANCE Trial,”^1^ published June 29, 2026, there were numeric errors in absolute risk differences in the Abstract and Results, an error in Results, a test name error in Statistical Analysis, number and percentage errors in the Table and the Supplement 1 eTable, and numeric errors in differences in Tables 2 and 3. This article has been corrected.^1^
